# Scoparone affects lipid metabolism in primary hepatocytes using lipidomics

**DOI:** 10.1038/srep28031

**Published:** 2016-06-16

**Authors:** Aihua Zhang, Shi Qiu, Hui Sun, Tianlei Zhang, Yu Guan, Ying Han, Guangli Yan, Xijun Wang

**Affiliations:** 1 Sino-US Chinmedomics Technology Cooperation Center, National TCM Key Laboratory of Serum Pharmacochemistry, Chinmedomics Research Center of TCM State Administration, Laboratory of Metabolomics, Heilongjiang University of Chinese Medicine, Heping Road, Harbin, China; 2 Department of Pharmaceutical Analysis, Heilongjiang University of Chinese Medicine, Harbin, China; 3 State Key Laboratory of Quality Research in Chinese Medicine, Macau University of Science and Technology, Avenida Wai Long, Taipa, Macau

## Abstract

Lipidomics, which focuses on the global study of molecular lipids in biological systems, could provide valuable insights about disease mechanisms. In this study, we present a nontargeted lipidomics strategy to determine cellular lipid alterations after scoparone exposure in primary hepatocytes. Lipid metabolic profiles were analyzed by high-performance liquid chromatography coupled with time-of-flight mass spectrometry, and a novel imaging TransOmics tool has been developed for the analysis of high-resolution MS data, including the data pretreatment, visualization, automated identification, deconvolution and quantification of lipid species. Chemometric and statistical analyses of the obtained lipid fingerprints revealed the global lipidomic alterations and tested the therapeutic effects of scoparone. Identification of ten proposed lipids contributed to the better understanding of the effects of scoparone on lipid metabolism in hepatocytes. The most striking finding was that scoparone caused comprehensive lipid changes, as represented by significant changes of the identificated lipids. The levels of identified PG(19:1(9Z)/14:0), PE(17:1(9Z)/0:0), PE(19:1(9Z)/0:0) were found to be upregulated in ethanol-induced group, whereas the levels in scoparone group were downregulated. Lipid metabolism in primary hepatocytes was changed significantly by scoparone treatment. We believe that this novel approach could substantially broaden the applications of high mass resolution mass spectrometry for cellular lipidomics.

Lipids play an outstanding role in diverse biological functions, including structural, energetic, and regulatory functions^1^. Numerous studies have demonstrated that disruption of lipid metabolism is associated with the onset and progression of many human diseases^2^,^3^. Identification and quantification of lipids could be of great interest to obtain in-depth knowledge of lipids function in the development of diseases. Lipidomics provides insights into the specific roles of lipid molecular species^4^. An effective lipidomics workflow is shotgun lipidomics, would ultimately produce a comprehensive dataset of MS and MS/MS spectra, providing capabilities for rapid and sensitive monitoring of the molecular compositions and abundances of lipids^5^. High-performance liquid chromatography coupled with time-of-flight mass spectrometry substantially broadened the analytical range, enabling the identification of isotope incorporation into intact lipids by MS/MS^6^. A major benefit of this technology is that hundreds of lipids can be directly identified and accurately quantified from total lipid extracts in a relatively short analysis time.

Alcohol abuse and alcoholism lead to alcoholic liver disease (ALD) that is one of the most important public health problems worldwide. ALD is a multifaceted disease that is characterized by a broad spectrum of liver injury^6^. Additionally, it increases the economic burden on society^7^. The liver is the main organ responsible for ethanol metabolism and therefore particularly prone to ethanol toxicity. Ethanol metabolism leads to alterations in the hepatic metabolism of lipids that subsequently produce a wide spectrum of hepatic injury^8^. Although greater efforts have been spent on the understanding of the mechanism, however, the cellular molecular lipid details for the metabolic functions in the ethanol-injured hepatic injury are not yet completely understood. Currently, there are three medications for the treatment of alcohol abuse and dependence that have been approved by the US FDA: disulfiram, naltrexone and acamprosate^9^. However, they have some side effects including gastrointestinal problems^10^. Many natural products, which have fewer side effects, have preventive and therapeutic effects for ethanol intoxication.

Scoparone (Fig. S1), a plant-derived coumarin, has antioxidative and hepatoprotective effects^11^. It was absorbed into the blood circulation within a few minutes, and the time to peak was at ~1 h after oral administration^12^. We previously reported that scoparone could protect the liver from ethanol-mediated hepatotoxicity and carbon tetrachloride-induced liver damage in experimental rats^13^,^14^. However, limited information is available regarding the changes in “global” lipid metabolism profiles of the hepatoprotective effects of scoparone. Here we firstly present a nontargeted lipidomics strategy to determine intracellular lipid alterations of scoparone against ethanol-induced cells. Lipids were extracted from exposed cells and analyzed by Ultra Performance Liquid Chromatography (UPLC)-Time of flight (TOF)-MS in full scan mode, together with imaging supported by TransOmics tool. The novel strategy presented here has the potential to open new detailed insights into the lipid metabolism that may lead to a better understanding of physiological mechanisms and metabolic perturbations, and has the potential to impact on drug discovery and development.

## Results

### Lipid analyses by UPLC-MS

**Figure 1:**
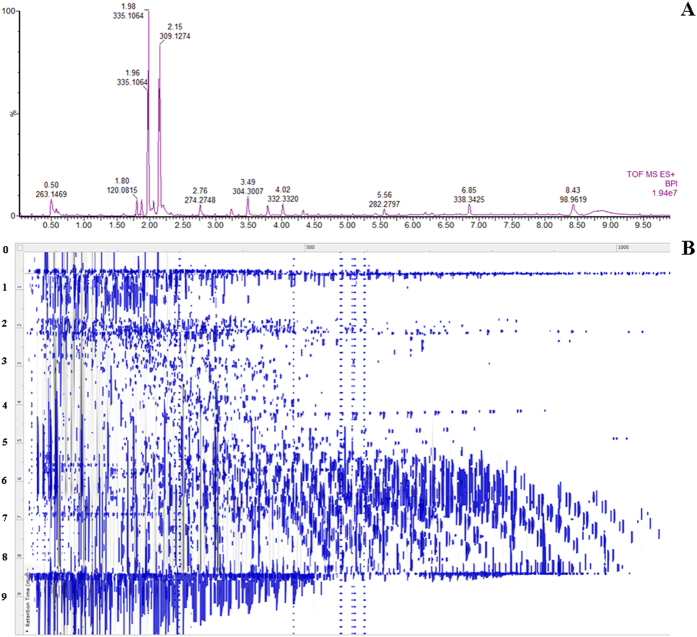
UPLC-MS analyses of lipid extracts from primary hepatocytes. Note: UPLC-MS base peak chromatogram chromatogram in positive ion mode (**A**); ‘compound ions’ in the 2D ion intensity map (**B**).

Lipids fraction were analyzed by LC-MS that performed the best profile in terms of peaks, symmetry, and resolution in reversed-phase fashion. To ensure repeatability of the analysis, the QC samples were randomly inserted among the real sample queue to be analyzed. Taking the results obtained from the cellular samples, RSD (relative standard derivations) of retention time and peak area was less than 0.96%, and 4.02%, respectively (Table S1). All the results indicated that the constructed method was robust with good repeatability and stability. Typical base peak ion (BPI) chromatograms of the cellular samples on UPLC-MS are shown in Fig. 1A and Fig. S2A. The alignment algorithm will generate ‘compound ions’ in the 2D ion intensity map, which consisted of approximately 5120 ion peaks (Fig. 1B) in positive ion mode and 3175 ion peaks (Fig. S2B) in negative ion mode from cellular UPLC-MS data. Panel B shows the two-dimensional map of retention time versus *m/z* of lipid species measured by a full scan within *m/z* 50–2000 in the positive ion mode.

### Lipid metabolic profiling analysis

**Figure 2:**
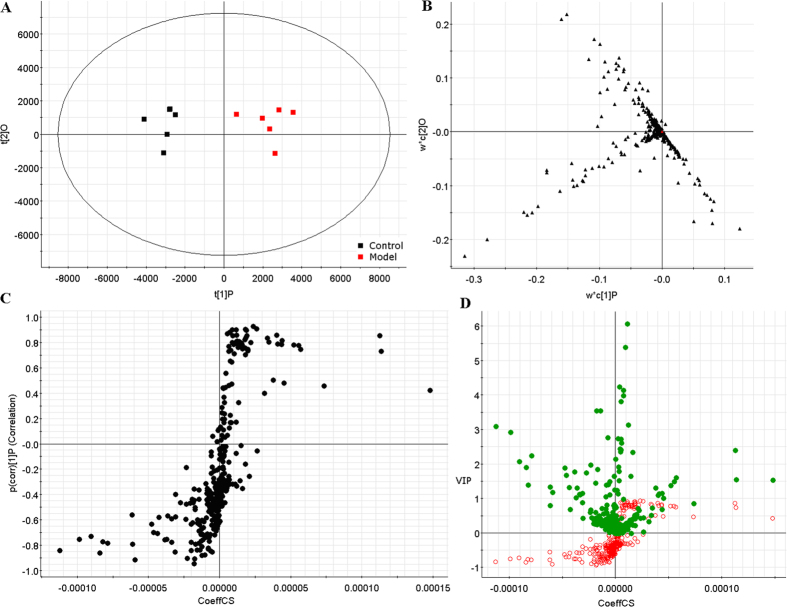
Multivariate analyses from cellular lipids in primary hepatocytes ion positive mode. (**A**) PCA score plot classifying the control and model groups from cellular lipids in positive ion mode. (**B**) Loading plot of PLS-DA model of LC-MS spectra data from intracellular lipids in positive ion mode. S-plot (**C**) and VIP-plot (**D**) of OPLS-DA model of LC-MS spectra data from intracellular lipids in positive ion mode.

**Figure 3:**
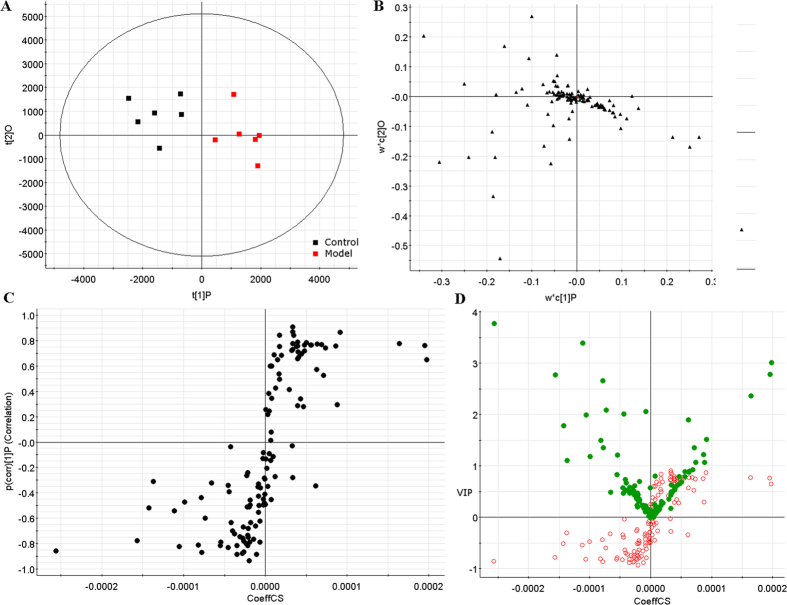
Multivariate analyses from cellular lipids in primary hepatocytes ion negative mode. (**A**) PCA score plot of the lipid profile classifying the control and model groups from cellular lipids in negative ion mode. (**B**) Loading plot of PLS-DA model of LC-MS spectra data from intracellular lipids in negative ion mode. S-plot (**C**) and VIP-plot (**D**) of OPLS-DA model of LC-MS spectra data from intracellular lipids in negative ion mode.

In order to identify the molecular species of lipids from cells, the UPLC-MS was applied along with “untargeted” (discovery profiling) approaches for lipidomics research. Raw data from UPLC-MS were analyzed by the TransOmics and then imported into EZinfo 2.0 software for multivariate data analysis. PCA scores plot showed clear separation between the control group and model group in both positive ion mode (Fig. 2A) and negative ion mode (Fig. 3A). This result suggested that cells displayed distinct biochemical perturbation characteristics under ethanol treatment. To see whether the differentially expressed lipids were accountable for the separation between control group and model group, a more sophisticated PLS-DA was carried out on the UPLC-MS data sets (Fig. 2B in positive ion mode and Fig. 3B in negative ion mode). From the corresponding the loading-plots, the ions furthest away from the origin may be therefore regarded as the differentiating lipids. The subsequent S-plots are used to evaluate the causative factors which result in different clustering on score plots (Fig. 2C in positive ion mode and Fig. 3C in negative ion mode). To select potential markers worthy of preferential study, lipids that differed statistically significantly between the control group and model group after correction for multiple comparisons (false discovery rate q < 0.05) were identified. Variables that significantly contributed to the clustering and discrimination were identified according to a threshold of variable importance in the projection (VIP) values (Fig. 2D in positive ion mode and Fig. 3D in negative ion mode), which could be generated after OPLS-DA processing. Following the criterion above, 10 significantly changed lipids (Table S2) which are linked to ethanol-induced primary hepatocytes were identified in intracellular lipidome.

### Identification and visualization of lipids

**Figure 4:**
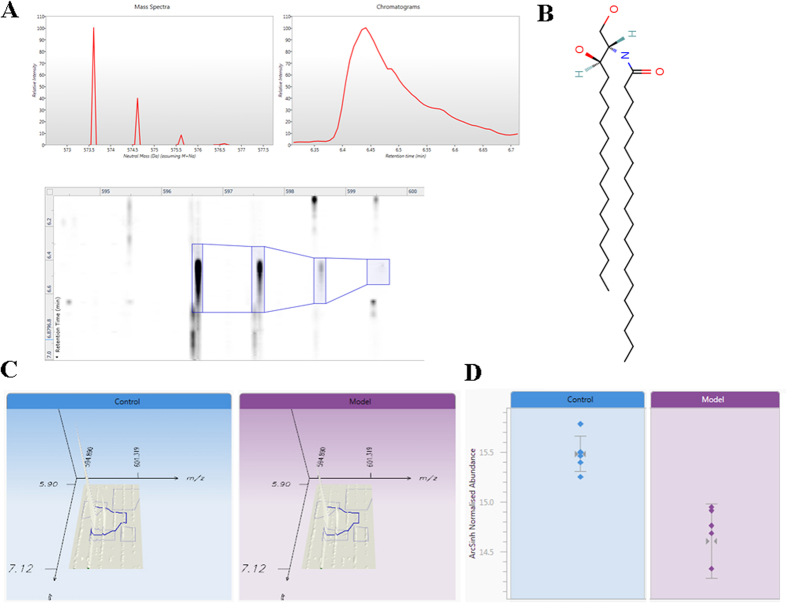
Identification and visualization of lipids. (**A**) Extracted ion chromatogram and MS/MS analysis on the lipid; (**B**) the molecular structure of Cer(d18:0/20:0); (**C**) the overexpressed lipid; (**D**) the relative intensity of the signal in control and model samples.

Lipid Map database was employed to carry out a tentative identification of the marker ions using their accurate mass measured by Q-TOF platform. From a total of 10 differential signals were tentatively identified. As shown in Table S2, mass error (ppm) obtained in the lipids identification, exceeded the 5 ppm found from the reference compound. In tandem mass spectrometric analyses, we could observe the fragment ions relating to their polar heads, adduct ions, and fatty acids. Among the total features significantly different in lipidome, the ion at *m/z* 596.5969 was taken as example for molecular confirmation by MS/MS fragmentation analysis. We performed MS/MS analysis on this lipid and, as shown in Fig. 4A, the fragments of the lipid are in agreement with the molecular structure of cer(d18:0/20:0) (Fig. 4B). This lipid eluted at 6.44 min and resulted in overexpressed intracellular lipidome as shown in Fig. 4C where the chromatographic peak and the relative intensity of the signal in control and model samples are reported in Fig. 4D. The selected exact mass for the lipids identification were detected using UPLC-MS (Fig. S3).

### Metabolic changes after scoparone exposure in cells

**Figure 5:**
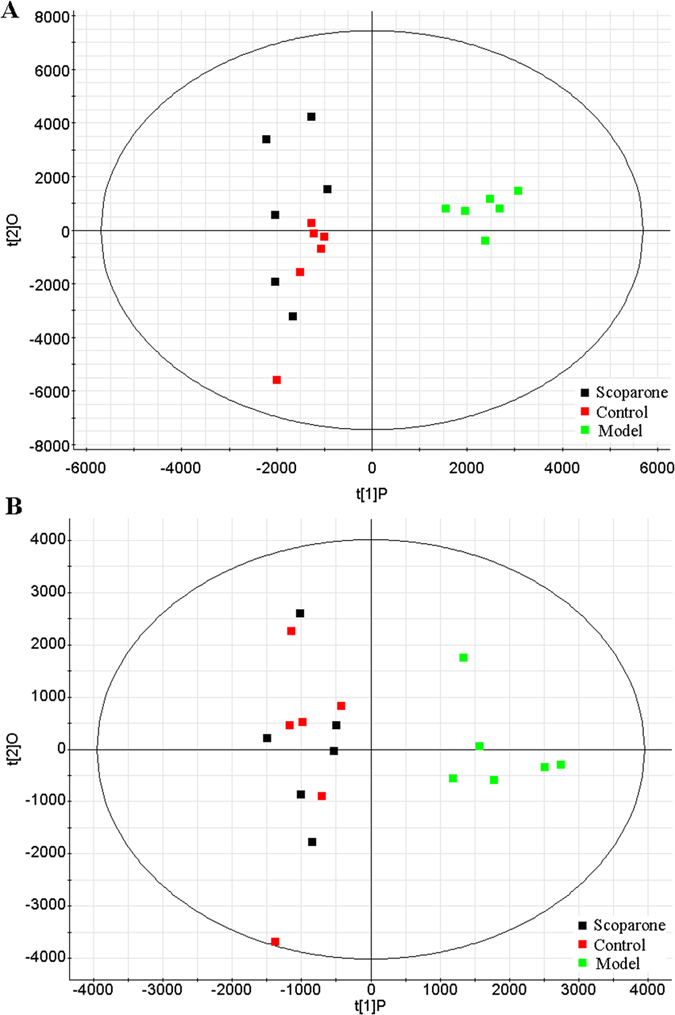
Lipid metabolic profiling changes caused by scoparone. (**A**) PCA score plot of cellular lipidome for classifying the control, model and scoparone group in positive ion mode; (**B**) PCA score plot of cellular lipidome for the clustering of control, model and scoparone group in negative ion mode.

**Figure 6:**
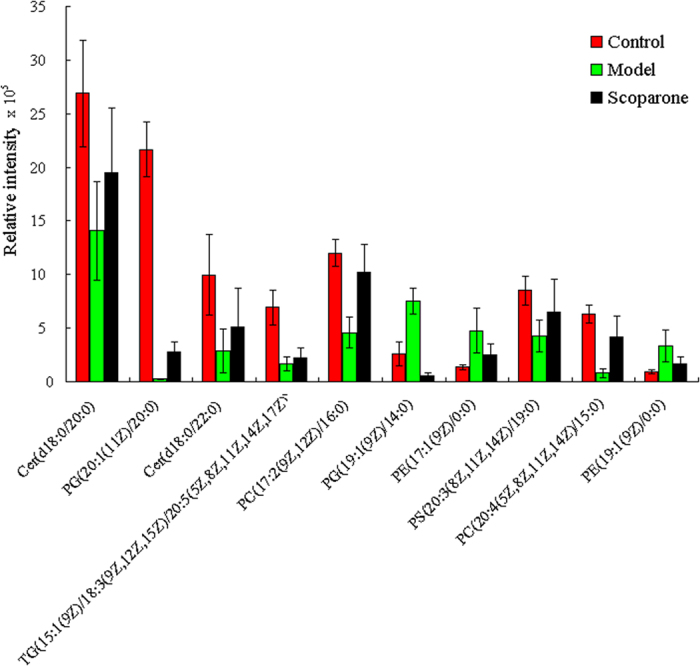
The relative levels of metabolites in cells after scoparone exposure. *Indicates significant difference when the values were compared to that of the control (p < 0.05). All tests were performed in triplicate and presented as means ± SE.

The pure standard of scoparone was tested on cells to study their cytotoxicity. Scoparone at 1.8 μg/mL exhibited none cytotoxic activity almost comparable to the control group (Fig. S3A). To determine the protective effects of scoparone, we studied the viability of primary hepatocytes that were treated with 1.8 μg/mL scoparone for 24 h and cultured under ethanol condition. We showed that treatment with scoparone effectively prevented the loss of cell viability induced by ethanol (Fig. S3B). From the PCA score plots (Fig. 5A,B) of cellular data, it can be seen that ethanol induced metabolic perturbations in primary hepatocytes. A definitive clustering among the control and scoparone samples can be seen. It possesses more variation from the scoparone treatment as compared to the ethanol and due to the fact that the metabolic changes upon cells exposure to scoparone. Interestingly, the abundances for most lipids were found to be regulated for treatment with scoparone (Fig. 6). Ten lipids changed significantly under scoparone intervention, including cer(d18:0/20:0), cer(d18:0/22:0), PG(20:1(11Z)/20:0), PG(19:1(9Z)/14:0), PC(17:2(9Z,12Z)/16:0), PC(20:4(5Z,8Z,11Z,14Z)/15:0), PE(17:1(9Z)/0:0), PE(19:1(9Z)/0:0), PS(20:3(8Z,11Z,14Z)/19:0), and TG(15:1(9Z)/18:3(9Z,12Z,15Z)/20:5(5Z,8Z, 11Z,14Z,17Z)) (Fig. 6).

## Discussion

Lipids, the fundamental components of biological membranes, are structurally and functionally a diverse class of metabolites, and play diverse and important roles in biological system^15^. Lipidomics has emerged as a crucial component in the broader push to arrive at an integrated picture of the role of genes, proteins, and metabolites that fully describes cellular function^16^,^17^. Alcohol abuse and alcoholism lead to ALD that is one of the most important public health problems worldwide. We previously showed that scoparone exhibited hepatoprotective action, yet the cellular lipid mechanisms of scoparone against the ALD are not well understood. In order to address the ethanol-mediated alterations in the cellular lipid profile, to elucidate the corresponding pattern, and to further reveal the underlying mechanisms as well as intracellular lipid alterations of scoparone against ethanol-induced primary hepatocytes, we performed a untargeted lipidomics investigation using the primary hepatocytes as a model system. To obtain the information about lipid variations underlying the treatment of scoparone, we simultaneously analyzed the intracellular lipidome in primary hepatocytes.

Owing to the high sensitivity and resolution for characterization of intact lipid species, LC-MS (ESI) can provide the most comprehensive and informative data set for cellular lipidome studies^18^. Examining the lipidome to assess drug candidates, we firstly conducted a comprehensive investigation on scoparone against ethanol-induced intracellular lipid changes in primary hepatocytes using LC-MS based untargeted lipidomics approach. From PCA scores plot, it suggested that cells displayed distinct metabolic characteristics under ethanol treatment. A total of 10 significantly changed lipids which are linked to ethanol-induced primary hepatocytes were identified in intracellular lipidome. From the PCA score plots, it is clear that it possesses more variation from the model as compared to the other time points and could be due to the fact that the metabolic changes upon exposure to scoparone. Of interest, PCA score plot shows a definitive clustering among the control and scoparone samples. Interestingly, the abundances for the lipids were found to be regulated for treatment with scoparone. Ten lipids changed significantly under scoparone intervention, including Cer(d18:0/20:0), PG(20:1(11Z)/20:0), Cer(d18:0/22:0), TG(15:1(9Z)/18:3(9Z,12Z,15Z)/20: 5(5Z,8Z,11Z,14Z,17Z)), PC(17:2(9Z,12Z)/16:0), PG(19:1(9Z)/14:0), PE(17:1(9Z)/0:0), PS(20:3(8Z,11Z,14Z)/19:0), PC(20:4(5Z,8Z,11Z,14Z)/15:0), and PE(19:1(9Z)/0:0). The levels of identified PG(19:1(9Z)/14:0), PE(17:1(9Z)/0:0), PE(19:1(9Z)/0:0) were found to be upregulated in ethanol-induced group, whereas the levels in scoparone group were downregulated. The levels of identified cer(d18:0/20:0), PG(20:1(11Z)/20:0), cer(d18:0/22:0), TG(15:1(9Z)/18:3(9Z,12Z,15Z)/20: 5(5Z,8Z,11Z,14Z,17Z)), PC(17:2(9Z,12Z)/16:0), PS(20:3(8Z, 11Z,14Z)/19:0), and PC(20:4(5Z,8Z,11Z,14Z)/15:0) were found to be downregulated in ethanol-induced group, whereas the levels in scoparone group were upregulated. These results suggest that scoparone ameliorates the ethanol-mediated injury in primary hepatocytes. Taken together, our data show for the first time how the cell lipids are affected by ethanol-induced states and provide a preliminary insight into the corresponding mechanisms.

Lipidomics represents the global identification and quantitative assessment of the vast array of lipids that play critical roles as mediators in energy homeostasis, membrane structure, and signaling. In this study, UPLC-TOF-MS method was developed for exhaustive lipid fingerprinting of primary hepatocytes samples, in full scan mode, together with imaging supported by TransOmics tool. PCA was used to investigate the global lipidomic alterations and to evaluate the therapeutic effects of scoparone. Identification of ten lipids contributed to the better understanding of the effects of scoparone on lipid metabolism in primary hepatocyte, as represented by significant changes of the identificated lipids. Overall, this work demonstrates the potential of incorporating UPLC-TOF-MS approach into cellular lipidomics research.

## Conclusion

In this work, we firstly present a nontargeted lipidomics strategy to determine intracellular lipid alterations of scoparone against ethanol-induced primary hepatocytes. Lipids from exposed cells were ultrasound extracted and analyzed by UPLC-TOF-MS in full scan mode with imaging TransOmics tool. Identification of 10 proposed lipids contributed to the better understanding of the effects of scoparone on lipid metabolism in hepatocytes. The levels of identified cer(d18:0/20:0), cer(d18:0/22:0), PG(20:1(11Z)/20:0), TG(15:1(9Z)/18:3(9Z,12Z,15Z)/20:5(5Z,8Z,11Z,14Z,17Z)), PC(20:4(5Z,8Z,11Z, 14Z)/15:0), PC(17:2(9Z,12Z)/16:0), and PS(20:3(8Z,11Z,14Z)/19:0), were found to be downregulated in ethanol-induced group, whereas the levels in scoparone group were upregulated. It showed that scoparone exposure at a low, non-cytotoxic dose can significantly alter metabolism in primary hepatocytes, thus providing the first evidence that scoparone may have biological effects on liver cells at realistic concentrations. The most striking finding was that scoparone caused comprehensive lipid changes, as represented by significant changes of the identificated lipids.

## Materials and Methods

### Chemicals

Ultrapure water, used to prepare all the aqueous solutions, was obtained from a Milli-Q system (Milipore, Bedford, MA, USA). Cell culture reagents were purchased from Sigma Chemical (Sigma, USA). Methanol and acetonitrile are chromatography pure (Merck, Germany). Lipid standards with purity >99% were purchased from Sigma-Aldrich (St Louis, MO). Stock solutions were prepared by dissolving lipid standards in MeOH/CHCl_3_ (50:50 *v*/*v*) at a concentration of 25 mg/mL and stored at −20 °C. Working solutions were diluted 10 μg/mL prior to spiking studies depending on the experiment.

### Cell culture and scoparone treatment

Primary hepatocytes were isolated from 6-week-old ICR male mice by two-step collagenase perfusion^19^. The freshly harvested hepatocytes were cultured in DMEM (supplemented with 10% fetal bovine serum, 32 IE/L insulin, 15 mM HEPES, 0.1 μM hydrocortisone, 100 U/mL penicillin and 100 U/mL streptomycin) in an incubator at 37 °C with 5% CO_2_. Cells were seeded in 24 wells-plates adding per well 1 mL cell suspension containing 1 × 10^5 ^cells/mL medium. We used 200 mM ethanol and 24 h exposure time as described previously. After 24 h, the medium was renewed and supplemented with ethanol (200 mM) for the following 24 h culture without serum (model group). After 24 h, the medium was refreshed, and scoparone group dissolved in DMSO were added at a final concentration of 1.8 μg/mL for 24 h and the final concentration of the solvent carrier DMSO was 0.1%, or an equal volume of DMSO as a control group. All cells were maintained in a 37 °C incubator with 5% CO_2_. After 24 h of exposure, the cells were washed with cold PBS 3 times, then stored at −80 °C until the lipid-extraction step. And then, the cells and supernatant were collected for various bioassays according to the corresponding experimental protocol. Cell viability was over 90% (trypan blue exclusion test) under these conditions.

### Lipid extraction

Extraction of lipids was performed to extract a broad lipidtype spectrum using a modified Folch method, as previously described^20^. The ultrasound frequency and power were 40 kHz and 20 W for 3 min, respectively, and temperature and time conditions were controlled for each analysis. The lipid extracts contained in the upper phase were collected and poured into an autosampler vial. The supernatants were then centrifuged at 4 °C for 10 min at 13,000 g. The broad lipid profile was determined.

### Liquid Chromatography Mass spectrometry Analysis (LC-MS)

LC-MS analysis was performed on a hybrid UPLC-MS consisting of a pump and autosampler equipped with an ACQUITY BEH C_18_ chromatography column with 0.17-μm stationary phase. The column temperature was maintained at 45 °C, and then gradient mobile phase was composed of phase A (acetonitrile containing 0.1% formic acid) and phase B (water with 0.1% formic acid). The following gradient was applied: 0–1 min, 1–55 % A; 1–5 min, 55–100 % A; 5–7 min, 100 % A isocratic; 7–7.5 min, 100–1 % A; 7.5–10 min, 1 % A isocratic. The injection volume was 2 μL and flow rate was 0.4 mL/min. A quality control (QC) sample was prepared by mixing 10 μL of each ample ready for injection. This QC sample is considered to be a representative sample containing all analytes that was therefore applied to condition the column before analysis. All samples were maintained at 4 °C during the analysis.

All MS spectra of the cell extracts were acquired on MS spectrometer (Waters Corp., Milford, USA) equipped with an electrospray ionization source that operates in positive ionization mode (ESI^+^) and negative ionization mode (ESI^−^) at 50–2000 *m/z* in the full scan mode. Nitrogen was used as the drying and collision gas. All the source and analyser parameters were optimized using MassLynx Optimizer software (Waters Corp., Milford, USA), respectively. The source parameters are as follows: capillary voltage 3000 V, cone voltage 20 V, desolvation temperature 350 °C, source temperature 110 °C, desolvation gas flow 600 L/h, and cone gas flow 50 L/h. Leucine enkaphalin was used as the reference compound at a concentration of 0.2 ng/mL under a flow rate of 100 μL·min^−1^ for accurate mass measurement. The analyser parameters are as follows: fragment or voltage 40 V, collision energy 15 V and cell accelerator voltage 10 V. Data were collected at a rate of 1 MS spectrum per second with a scan time of 0.4 s, an inter-scan delay of 0.1 s, and a lock spray frequency of 10 s. The collision energy was a function of the precursor ion, acquiring the MS/MS spectra by automatic switching between MS and MS/MS mode.

### Data processing

All the LC–MS raw files were converted to TransOmics program (Waters, Milford, USA) for data processing was used considering inclusion criteria compatible with analytical performance obtained (mass accuracy, precision, and retention time reproducibility). This procedure allowed peak picking, deconvolution, alignment, and data reduction to give a table of mass and retention time pairs with associated relative intensities for all the detected peaks. The normalized data were used for multivariate analysis, and the model was constructed using the unsupervised principal components analysis (PCA) and supervised partial latent structures-discriminant analysis (PLS-DA), orthogonal projection to latent structure-discriminant analysis (OPLS-DA) by EZinfo software (Waters Corp., Milford, USA). The combining VIP-plot (>2) from the OPLS analysis were carried out to select distinct variables as potential markers. Lipid identification and annotation were based on a combination of database query using exact mass measurement (mass error <5 ppm) and MS/MS pattern measured by Q-TOF platform. The databases used were Metlin, LIPID MAPS and Human Metabolome databases.

### Statistical analysis

All the LC-MS raw files were converted to TransOmics software (Waters, Millford, MA, USA). Differences in the amounts of molecules between groups were analysed using SPSS Statistics 19.0 (SPSS, Chicago, IL, USA). The p values less than 0.05 were considered significant.

## Additional Information

**How to cite this article**: Zhang, A. *et al*. Scoparone affects lipid metabolism in primary hepatocytes using lipidomics. *Sci. Rep.* **6**, 28031; doi: 10.1038/srep28031 (2016).

## Supplementary Material

Supplementary Information

## References

[b1] Maeda K. . Interactome map uncovers phosphatidylserine transport by oxysterol-binding proteins. Nature 501, 257–261 (2013). 23934110 10.1038/nature12430

[b2] Liang C. . Aptamer-functionalized lipid nanoparticles targeting osteoblasts as a novel RNA interference-based bone anabolic strategy. Nat Med 21, 288–294 (2015). 25665179 10.1038/nm.3791 PMC5508976

[b3] Flach T. L. . Alum interaction with dendritic cell membrane lipids is essential for its adjuvanticity. Nat Med 17, 479–387 (2011). 21399646 10.1038/nm.2306

[b4] Wang C. . Comprehensive and quantitative analysis of lysophospholipid molecular species present in obese mouse liver by shotgun lipidomics. Anal Chem 87, 4879–4887 (2015). 25860968 10.1021/acs.analchem.5b00410 PMC4657943

[b5] Kenar E. . Automated label-free quantification of metabolites from liquid chromatography-mass spectrometry data. Mol Cell Proteomics 13, 348–359 (2014). 24176773 10.1074/mcp.M113.031278 PMC3879626

[b6] Whelan R. . Neuropsychosocial profiles of current and future adolescent alcohol misusers. Nature 512, 185–189 (2014). 25043041 10.1038/nature13402 PMC4486207

[b7] DePoy L. . Chronic alcohol produces neuroadaptations to prime dorsal striatal learning. Proc Natl Acad Sci USA 110, 14783–14788 (2013). 23959891 10.1073/pnas.1308198110 PMC3767559

[b8] Kwon H. J. . Aldehyde dehydrogenase 2 deficiency ameliorates alcoholic fatty liver but worsens liver inflammation and fibrosis in mice. Hepatology 60, 146–157 (2014). 24492981 10.1002/hep.27036 PMC4077916

[b9] Mark T. L. . Alcohol and opioid dependence medications: prescription trends, overall and by physician specialty. Drug Alcohol Depend. 99, 345–349 (2009). 18819759 10.1016/j.drugalcdep.2008.07.018 PMC3166770

[b10] Addolorato G. . Management of alcohol dependence in patients with liver disease. CNS Drugs. 27, 287–299 (2013). 23456576 10.1007/s40263-013-0043-4 PMC4979989

[b11] Zhang A. . Proteomics analysis of hepatoprotective effects for scoparone using MALDI-TOF/TOF mass spectrometry with bioinformatics. OMICS. 17, 224–229 (2013). 23514563 10.1089/omi.2012.0064 PMC3615692

[b12] Yin Q. . Pharmacokinetics and tissue distribution study of scoparone in rats by ultraperformance liquid-chromatography with tandem high-definition mass spectrometry. Fitoterapia. 83, 795–800 (2012). 22465507 10.1016/j.fitote.2012.03.010

[b13] Zhang A., Sun H. & Wang X. Urinary metabolic profiling of rat models revealed protective function of scoparone against alcohol induced hepatotoxicity. Sci Rep. 4, 6768 (2014). 25341677 10.1038/srep06768 PMC4208028

[b14] Zhang A. . Metabolomics study on the hepatoprotective effect of scoparone using ultra-performance liquid chromatography/electrospray ionization quadruple time-of-flight mass spectrometry. Analyst. 138, 353–361 (2013). 23152956 10.1039/c2an36382h

[b15] Madigan C. A. . Lipidomic discovery of deoxysiderophores reveals a revised mycobactin biosynthesis pathway in Mycobacterium tuberculosis. Proc Natl Acad Sci USA 109, 1257–1262 (2012). 22232695 10.1073/pnas.1109958109 PMC3268331

[b16] Vorkas P. A. . Metabolic phenotyping of atherosclerotic plaques reveals latent associations between free cholesterol and ceramide metabolism in atherogenesis. J Proteome Res 14, 1389–1399 (2015). 25565173 10.1021/pr5009898

[b17] Li F. . Lipidomics reveals a link between CYP1B1 and SCD1 in promoting obesity. J Proteome Res 13, 2679–2687 (2014). 24684199 10.1021/pr500145n PMC4018097

[b18] Bollinger J. G. . LC/ESI-MS/MS detection of FAs by charge reversal derivatization with more than four orders of magnitude improvement in sensitivity. J Lipid Res 54, 3523–3530 (2013). 23945566 10.1194/jlr.D040782 PMC3826698

[b19] Wang H. . Post-isolation inducible nitric oxide synthase gene expression due to collagenase buffer perfusionand characterization of the gene regulation in primary cultured murine hepatocytes. J Biochem. 124, 892–899 (1998). 9792910 10.1093/oxfordjournals.jbchem.a022204

[b20] Nygren H. . Liquid chromatography-mass spectrometry (LC-MS)-based lipidomics for studies of body fluids and tissues. Methods Mol Biol 708, 247–257 (2011). 21207295 10.1007/978-1-61737-985-7_15

