# Supplementary material for: Scoparone affects lipid metabolism in primary hepatocytes using lipidomics
Source: Sci Rep. 2016 Jun 16;6:28031. doi: 10.1038/srep28031 (PMC4910084; doi:10.1038/srep28031)
Supplement: Supplementary Information [file srep28031-s1.doc]

**Scoparone affects lipid metabolism in primary hepatocytes using lipidomics**

Aihua Zhang^1,2^, Shi Qiu^1,2^, Hui Sun^1,3^, Tianlei Zhang^1,2^, Yu Guan^1,2^, Ying Han^1,2^, Guangli Yan^1,2^, Xijun Wang^1,2^*

^1^ National TCM Key Laboratory of Serum Pharmacochemistry, Laboratory of Metabolomics, Heilongjiang University of Chinese Medicine, Heping Road 24, Harbin 150040, China.

^2^ Research Center of Chinmedomics, Heilongjiang University of Chinese Medicine, Heping Road 24, Harbin 150040, China.

^3^ Department of Pharmaceutical Analysis, School of Pharmacy, Heilongjiang University of Chinese Medicine, Heping Road, Harbin, China.

*Correspondence

Prof. Xijun Wang

National TCM Key Laboratory of Serum Pharmacochemistry, Laboratory of Metabolomics,

Research Center of Chinmedomics, Heilongjiang University of Chinese Medicine, Heping Road, Harbin, China.

Tel. & Fax +86-451-82110818

Email: xijunwanglsls@yeah.net


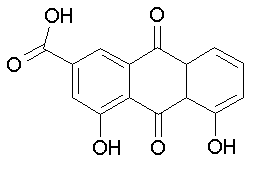


**Figure** **S1**. The chemical structure of scoparone.


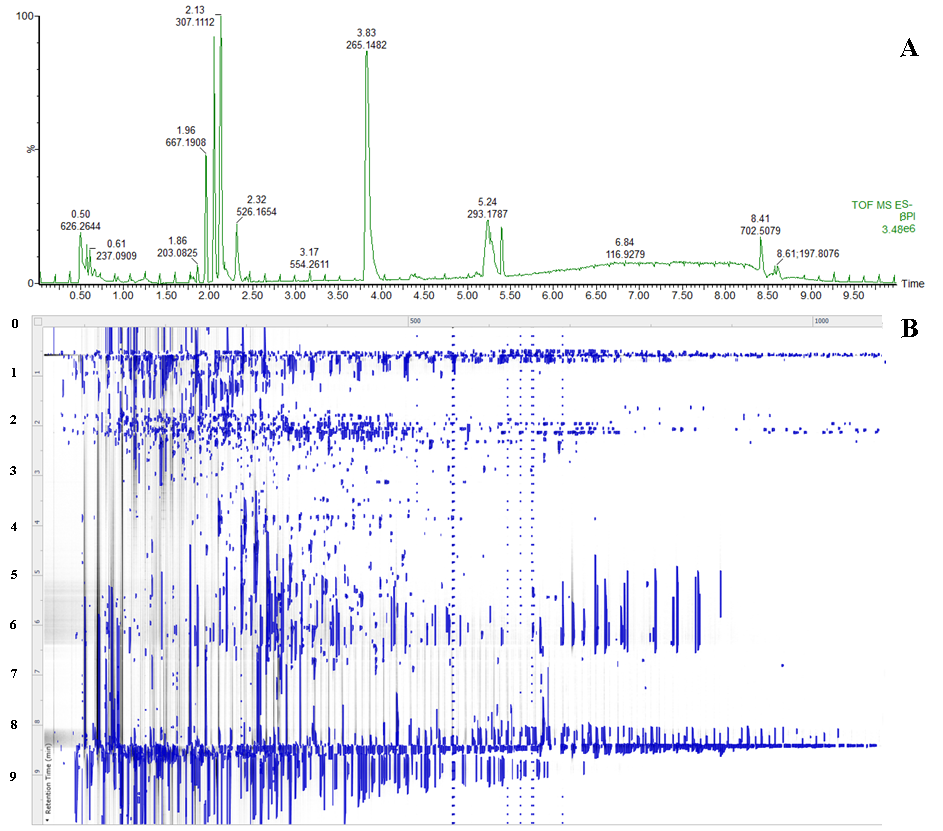


**Figure S2.** **Lipid analyses of primary hepatocytes by UPLC-MS**

Note: UPLC/MS BPI chromatogram in negative ion mode (**A**); ‘compound ions’ in the 2D ion intensity map (**B**)


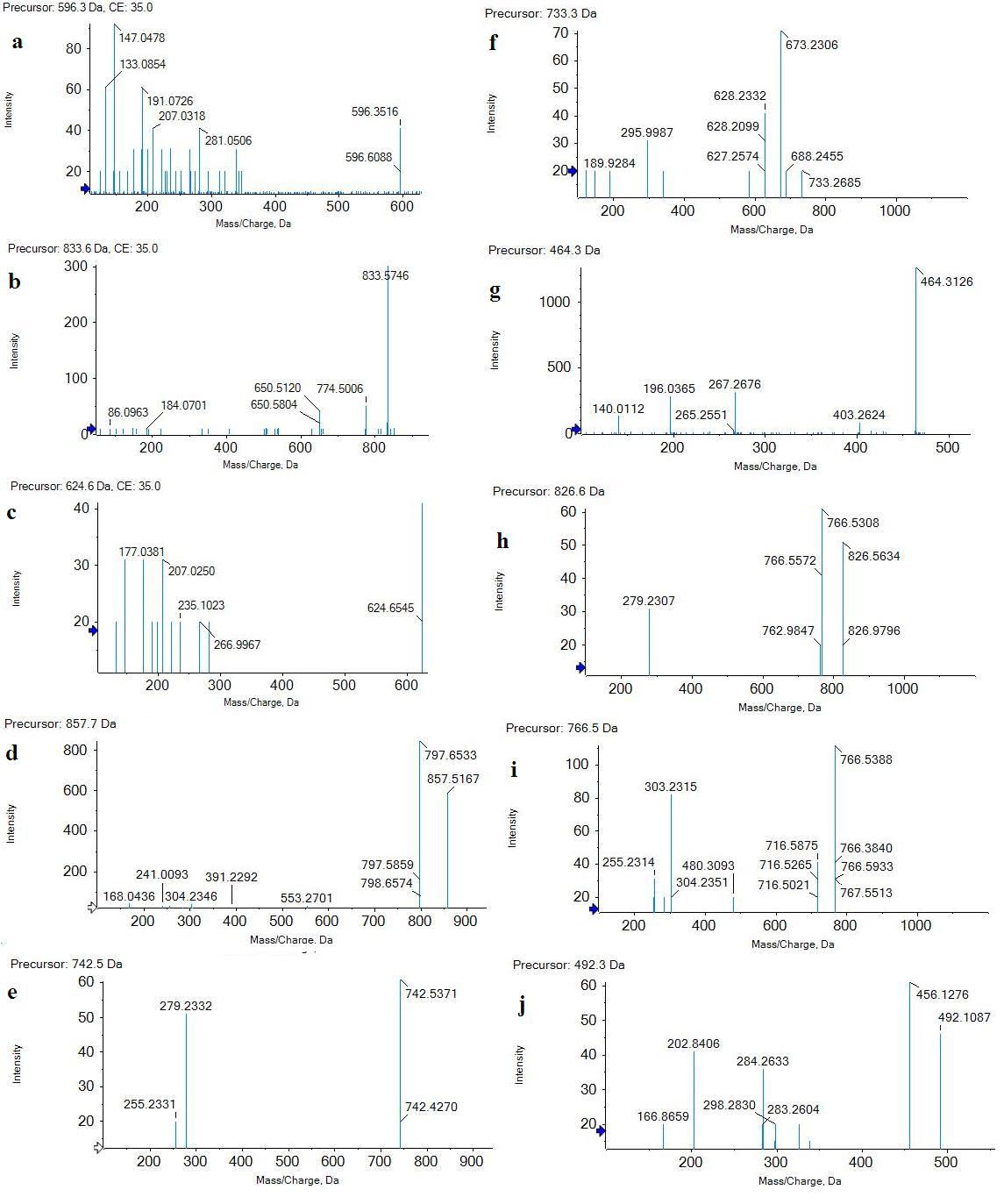


**Figure S3.** The selected exact mass for the lipids m/z. The LC and MS conditions are described in Table S2, respectively. a, Cer(d18:0/20:0); b, PG(20:1(11Z)/20:0); c, Cer(d18:0/22:0); d, TG(15:1(9Z)/18:3(9Z,12Z, 15Z)/20:5(5Z,8Z,11Z,14Z,17Z)); e, PC(17:2(9Z,12Z)/16:0); f, PG(19:1(9Z)/14:0); g, PE(17:1(9Z)/0:0); h, PS(20:3(8Z,11Z,14Z)/19:0); i, PC(20:4(5Z,8Z,11Z,14Z)/15:0); j, PE(19:1(9Z)/0:0).

**
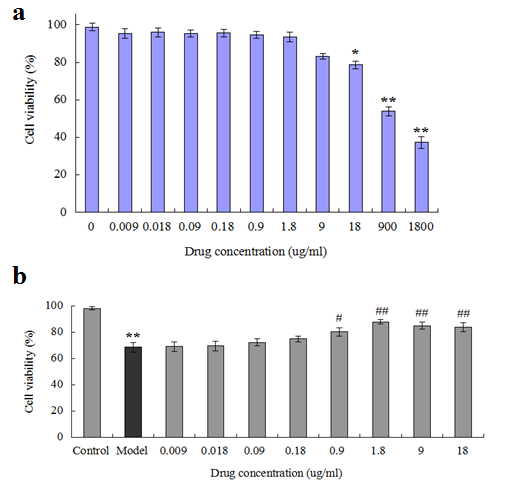
**

**Figure S4.** Effect of scoparone on ethanol-induced primary hepatocytes damages.

(**A**) Effect of scoparone on ethanol-induced cytotoxicity in cells. (**B**) Dose-dependent effects of scoparone and ethanol on cells after 24 h treatment. Data are presented as the means±S.D. for at least 3 independent experiments. *p< 0.05 and **p< 0.01 as compared with the control cells; #p< 0.05 and ##p<0.01 as compared with ethanol-treated model cells

**Table S1.** The result of stability and repeatability of the proposed UPLC-MS method

| **Mode** | ***m/z*** | **Stability (n=6)** | | | | **Repeatability (n=6)** | | | |
| --- | --- | --- | --- | --- | --- | --- | --- | --- | --- |
|  |  | **RT(min)** | | **Peak area** | | **RT(min)** | | **Peak area** | |
|  |  | **Mean** | **RSD(%)** | **Mean** | **RSD(%)** | **Mean** | **RSD(%)** | **Mean** | **RSD(%)** |
| **ESI+** | 256.26 | 1.43 | 0.34 | 4027 | 1.68 | 1.43 | 0.71 | 4118 | 2.12 |
|  | 338.34 | 2.65 | 0.96 | 11687 | 2.45 | 2.65 | 0.87 | 11534 | 1.73 |
|  | 261.13 | 3.23 | 0.05 | 113235 | 1.94 | 3.23 | 0.06 | 110017 | 1.30 |
|  | 380.32 | 3.79 | 0.21 | 13198 | 3.63 | 3.79 | 0.43 | 13385 | 3.59 |
|  | 289.63 | 4.56 | 0.60 | 1490 | 2.70 | 4.56 | 0.83 | 1435 | 2.08 |
| **ESI-** | 230.87 | 1.01 | 0.10 | 4203 | 4.02 | 1.01 | 0.20 | 4262 | 0.77 |
|  | 379.10 | 2.08 | 0.07 | 13103 | 3.51 | 2.08 | 0.04 | 13056 | 1.92 |
|  | 288.92 | 2.7 | 0.29 | 2273 | 3.18 | 2.7 | 0.37 | 2336 | 2.18 |
|  | 172.98 | 3.32 | 0.23 | 3181 | 1.64 | 3.32 | 0.18 | 2990 | 1.42 |
|  | 166.93 | 4.11 | 0.24 | 1102 | 2.37 | 4.11 | 0.23 | 1044 | 3.85 |

**Table S2. Detailed MRM conditions and retention times of lipids detected using UPLC-MS**

| **Name** | **tR (min)** | ***m/z*** | **Compound ID** | **Ion**  **mode** | **Formula** | **Mass**  **Error**  **(ppm)** | **Dwell**  **Time**  **[ms]** | **Collision**  **energy**  **[V]** | **Anova (p)** | **Max Fold**  **Change** | **VIP** |
| --- | --- | --- | --- | --- | --- | --- | --- | --- | --- | --- | --- |
| Cer(d18:0/20:0) | 6.44 | 596.60 | LMSP02020009 | Positive | C38H77NO3 | -1.20 | 5 | 35 | 0.0043 | 2.09 | 3.10 |
| PG(20:1(11Z)/20:0) | 7.56 | 833.63 | LMGP04010547 | Positive | C46H89O10P | 3.97 | 5 | 35 | 0.0217 | 549.34 | 2.44 |
| Cer(d18:0/22:0) | 6.91 | 624.63 | LMSP02020010 | Positive | C40H81NO3 | -4.98 | 5 | 35 | 0.0114 | 3.46 | 2.25 |
| TG(15:1(9Z)/18:3(9Z,12Z,15Z)/20:  5(5Z,8Z,11Z,14Z,17Z)) | 8.42 | 857.67 | LMGL03015562 | Negative | C56H90O6 | 2.05 | 5 | 35 | 0.0000 | 4.16 | 3.79 |
| PC(17:2(9Z,12Z)/16:0) | 6.22 | 742.54 | LMGP01011557 | Negative | C41H78NO8P | 0.11 | 5 | 35 | 0.0095 | 2.64 | 3.40 |
| PG(19:1(9Z)/14:0) | 6.10 | 733.51 | LMGP04010482 | Negative | C39H75O10P | 3.39 | 5 | 35 | 0.0051 | 13.59 | 3.02 |
| PE(17:1(9Z)/0:0) | 3.87 | 464.28 | LMGP02050008 | Negative | C22H44NO7P | -2.03 | 5 | 35 | 0.0008 | 3.48 | 2.79 |
| PS(20:3(8Z,11Z,14Z)/19:0) | 5.93 | 826.56 | LMGP03010606 | Negative | C45H82NO10P | 0.85 | 5 | 35 | 0.0473 | 2.00 | 2.78 |
| PC(20:4(5Z,8Z,11Z,14Z)/15:0) | 6.22 | 766.54 | LMGP01011901 | Negative | C43H78NO8P | 1.19 | 5 | 35 | 0.0204 | 7.99 | 2.67 |
| PE(19:1(9Z)/0:0) | 4.62 | 492.31 | LMGP02050019 | Negative | C24H48NO7P | -2.00 | 5 | 35 | 0.0005 | 3.62 | 2.37 |
